# Potential Role of JAK-STAT Signaling Pathway in the Neurogenic-to-Gliogenic Shift in Down Syndrome Brain

**DOI:** 10.1155/2016/7434191

**Published:** 2016-01-12

**Authors:** Han-Chung Lee, Kai-Leng Tan, Pike-See Cheah, King-Hwa Ling

**Affiliations:** ^1^NeuroBiology & Genetics Group, Genetics & Regenerative Medicine Research Centre, Faculty of Medicine and Health Sciences, Universiti Putra Malaysia (UPM), 43400 Serdang, Selangor, Malaysia; ^2^Medical Genetics Laboratory, Department of Biomedical Sciences, Faculty of Medicine and Health Sciences, Universiti Putra Malaysia (UPM), 43400 Serdang, Selangor, Malaysia; ^3^Department of Human Anatomy, Faculty of Medicine and Health Sciences, Universiti Putra Malaysia (UPM), 43400 Serdang, Selangor, Malaysia

## Abstract

Trisomy of human chromosome 21 in Down syndrome (DS) leads to several phenotypes, such as mild-to-severe intellectual disability, hypotonia, and craniofacial dysmorphisms. These are fundamental hallmarks of the disorder that affect the quality of life of most individuals with DS. Proper brain development involves meticulous regulation of various signaling pathways, and dysregulation may result in abnormal neurodevelopment. DS brain is characterized by an increased number of astrocytes with reduced number of neurons. In mouse models for DS, the pool of neural progenitor cells commits to glia rather than neuronal cell fate in the DS brain. However, the mechanism(s) and consequences of this slight neurogenic-to-gliogenic shift in DS brain are still poorly understood. To date, Janus kinase-signal transducer and activator of transcription (JAK-STAT) signaling has been proposed to be crucial in various developmental pathways, especially in promoting astrogliogenesis. Since both human and mouse models of DS brain exhibit less neurons and a higher percentage of cells with astrocytic phenotypes, understanding the role of JAK-STAT signaling in DS brain development will provide novel insight into its role in the pathogenesis of DS brain and may serve as a potential target for the development of effective therapy to improve DS cognition.

## 1. Introduction

It is well known that the presence of all or part of an extra copy of chromosome 21 (HSA21) causes Down syndrome (DS). The worldwide prevalence of DS is about 1 in 1000 live births [1]. An increased HSA21 copy number results in DS phenotypes, such as upward slanting eyes, flat facial features, and intellectual disability [2]. Individuals with DS also develop hypotonia, congenital heart defects, cognitive impairment, and early onset of the Alzheimer disease (AD). Approximately 50–70% of DS individuals develop dementia before the age of 60 [3]. The clinical features vary; however, intellectual disability remains an invariable hallmark of this syndrome and may be related to impairment of neurogenesis.

Individuals with DS demonstrate central nervous system abnormalities, such as reduced brain size, weight, volume, neuronal density, and neuronal distribution as well as increased synaptic abnormalities [4–10]. Studies to date using DS mouse models and aborted human DS fetuses have revealed defective cell proliferation and neurogenesis in several brain regions, such as the cerebellum and hippocampus [11, 12], which are critical for motor movement, learning, and memory. Notably, the reduced number of neurons is due to severely impaired proliferation of cerebellar cells and an increased number of apoptotic cells in the hippocampal region of human fetuses with DS. In contrast, the number of glial cells, especially astrocytes, has been shown to be increased in the DS brain [13, 14]. In the mammalian brain, astrocytes are the predominant cell type and are essential for regulating synapse formation [15], synaptic plasticity [16], maintaining the blood brain barrier, regulating neurotransmitters, and preserving ion homeostasis [17]. The consequences of gliogenic shift in the DS brain remain unknown. It has been postulated that the shift potentially causes neurogenesis and proliferation defects, which likely is due to reduction of neuronal precursor specification or overall cell-cycling speeds [18]. Therefore, a gliogenic shift in the DS brain may disturb homeostasis and affect the brain development, which may be a major factor contributing to the intellectual disability observed in DS individuals.

The discovery of neural progenitor cell bias towards glial lineages has been shown to be consistent in the brain of both human and mouse model for DS. In human DS fetuses, the percentage of astrocytes in the hippocampal region has been shown to be significantly higher compared to control fetuses [13]. A similar observation was also found in other brain regions, such as the frontal lobe of human DS fetuses [19]. In a DS mouse model, Contestabile and colleagues [14] also reported a comparable observation in Ts65Dn versus disomic mice where the number of cells with an astrocytic phenotype in the hippocampal dentate gyrus was larger in Ts65DN. Neurosphere cultures derived from Ts1Cje mouse models of DS further demonstrated a reduction in the number of neurons, whereas the number of astrocytes was increased [20]. Moreover, a twofold increase in the number of astrocytes derived from human DS-induced pluripotent stem cell (iPSC) cultures was also reported [18]. Recent evidence has shown that a gliocentric shift in DS astrocytes caused a reduction of neurogenesis and neuronal cell death via the release of S100B, which resulted in elevated nitric oxide (NO) generation [21]. Therefore, understanding the mechanism(s) underlying the neurogenic-to-gliogenic shift and the consequences in DS brain may shed light on the etiology of early neurodegeneration as well as neuronal reduction, which may contribute to the intellectual disability seen in individuals with DS.

The Janus kinase-signal transducer and activator of transcription (JAK-STAT) signaling pathway is one of the major gliogenic pathways [22]. Upon activation by gliogenic factor/cytokines, the JAK-STAT signaling pathway specifies glial differentiation. Importantly, genes encoding receptors for interferons (interferon-*α* receptor 1 (IFNAR1), IFNAR2, and IFN-*γ* R2 (IFNGR2)) responsible for activating JAK-STAT signaling cascades were found to be located on HSA21 and are triplicated in DS [23], suggesting a potential dysregulation of the downstream JAK-STAT signaling leading to activation of gliogenesis in DS brain. Herein, we review the association of IFNs with JAK-STAT signaling and highlight the potential role of this pathway in promoting the neurogenic-to-gliogenic shift in DS brain, which may lead to the development of novel therapeutics for DS.

## 2. The Canonical JAK-STAT Signaling Pathway

The first evidence of JAK-STAT signaling pathway involvement in brain development was based on primary cultures of embryonic cortical precursor cells derived from rats [22]. However, JAK-STAT signaling is not limited to brain development, as it is also involved in the development of hematopoietic cells and regulatory immune responses [24]. More importantly, normal function of this pathway is necessary for neural stem cell maintenance, growth, and renewal as well as overall cell survival and apoptosis [25].

In mammals, JAK proteins are comprised of four members (JAK1–3 and tyrosine kinase (TYK) 2), while the STAT proteins consist of seven (STAT1–4, STAT5a, STAT5b, and STAT6) [26]. The JAK-STAT signaling pathway is activated when various ligands bind to their corresponding receptor (Table 1). The receptors in the JAK-STAT pathway do not have tyrosine kinase activity and therefore are not able to activate any signaling cascades. JAK members initiate the signaling cascade by phosphorylating downstream transcription factors. In general, the receptor associates with JAK; the JAKs are brought into close proximity when ligands bind their corresponding receptor, leading to dimerization of receptor subunits and allowing the JAKs to phosphorylate each other [27]. Transphosphorylation results in JAK activation, which allows them to phosphorylate the receptor and create a binding site for SH2 domains of STATs [28].

**Figure 1:**
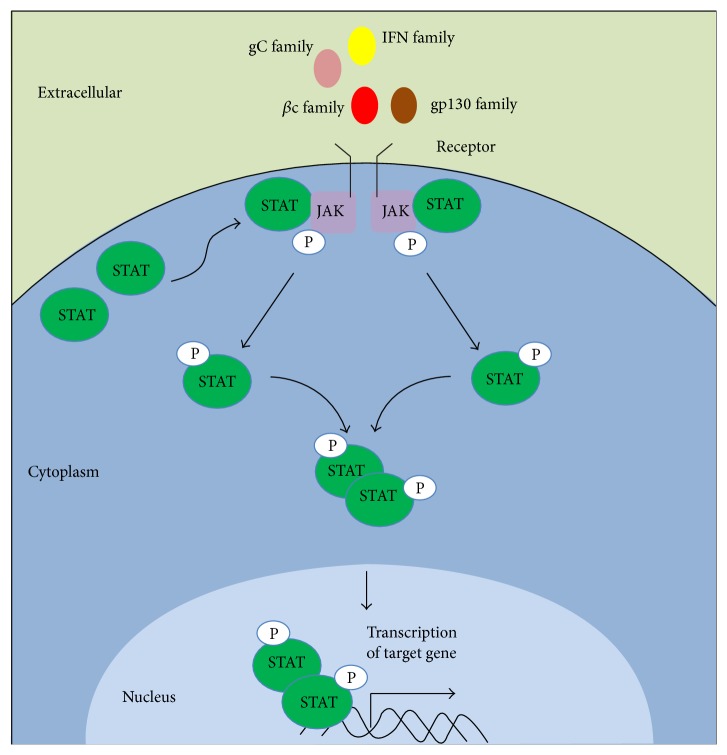
The canonical JAK-STAT signaling pathway. Ligands bind to corresponding receptors and allow the appropriate JAKs to phosphorylate each other, leading to their activation. Activated JAKs phosphorylate STATs, followed by subsequent dimerization of the STATs. STAT dimers are transported into the nucleus and bind the promoters of target genes to initiate transcription. The ligands include interleukins (IL-10, IL-19, IL-20, and IL-22), IFNs (IFN*α*, IFN*β*, and IFN*γ*), gp130 family (IL-6, IL-11, oncostatin M, leukemia inhibitory factor, cardiotrophin-1, granulocyte colony-stimulating factor, IL-12, Leptin, and ciliary neurotrophic factor), and *γ*-chain (gC) family (IL-2, IL-4, IL-7, IL-9, IL-15, and IL-21).

The STATs are latent transcription factors that reside in the cytoplasm but become activated when STATs bind to the receptor and JAKs phosphorylate the conserved Tyr residue near the C-terminus of STATs [28, 29]. Upon activation by Tyr phosphorylation, members of the STAT family then interact with each other and dimerize through their conserved SH2 domains. Consequently, phosphorylated STATs are transported from the cytoplasm into the nucleus. Once in the nucleus, dimerized STATs bind to the promoters of target genes to initiate transcription (Figure 1) [28]. The well-known downstream target of JAK-STAT signaling cascade is glial fibrillary acidic protein (*Gfap*), which is required for astrocyte differentiation [30].

## 3. Expression Patterns of JAK-STAT during Mouse Brain Development

**Figure 2:**
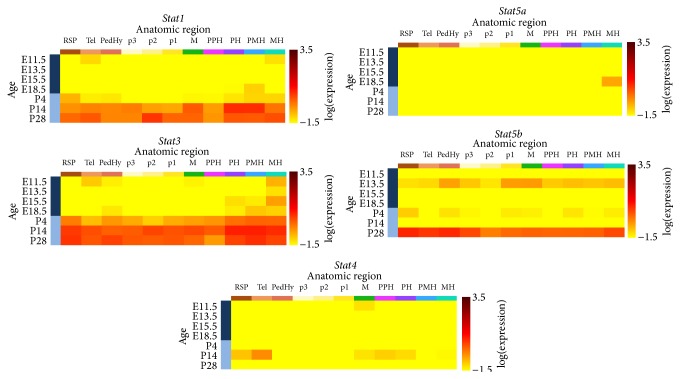
*Stat* (*Stat1*,* Stat3*,* Stat4*,* Stat5a*, and* Stat5b*) gene expression in C57BL/6J mouse whole brain throughout development presented in each anatomical region with log⁡ (expression).* Jak* gene expression data are not available. RSP: rostral secondary prosencephalon; Tel: telencephalic vesicle; PedHy: peduncular (caudal) hypothalamus; p3: prosomere 3; p2: prosomere 2; p1: prosomere 1; M: midbrain; PPH: prepontine hindbrain; PH: pontine hindbrain; PMH: pontomedullary hindbrain; MH: medullary hindbrain (medulla). Allen Developing Mouse Brain Atlas 2013 (http://developingmouse.brain-map.org/).

Messenger RNA expression of* Stat*s varies and is dependent on different brain developmental stages (Figure 2). Gene expression data mining from the Allen Developing Mouse Brain Atlas (http://developingmouse.brain-map.org/) showed low expression of* Stat1 *in all brain regions from embryonic day (E) 11.5 to postnatal day (P) 4 with a gradual increase in expression from P14 to P28.* Stat1* was highly expressed in the pontine and pontomedullary hindbrain at P14 and also prosomere 2 at P28 compared to other brain regions. Similar to* Stat1*,* Stat3 *was highly expressed between P4 and P28.* Stat4 *had low expression throughout all developmental stages, except the telencephalic vesicle region at P14 compared to other brain regions.* Stat5a *expression wasalso low throughout all developmental stages, except the medullary hindbrain region during E18.5. Although* Stat5b *exhibited generally low expression in all brain regions throughout development (E11.5–P14), it was found to be highly expressed only at P28 throughout various regions in the brain. No* in situ* hybridization of* Jak* and* Stat6* was found in the Allen Developing Mouse Brain Atlas.

**Figure 3:**
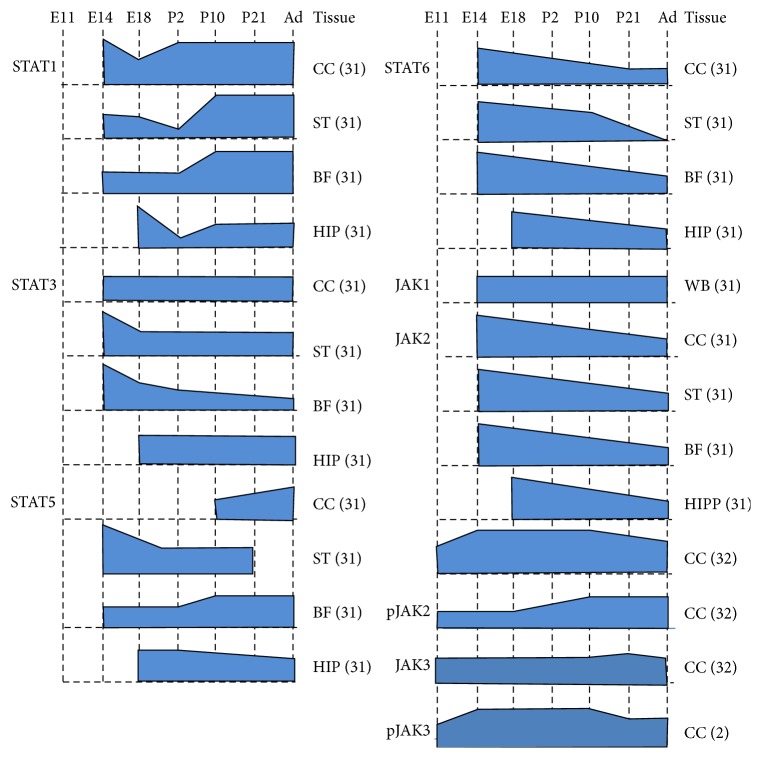
JAK (JAK1, JAK2, and JAK3), phosphorylated JAK (pJAK2 and pJAK3), and STAT (STAT1, STAT3, STAT5, and STAT6) protein levels in the mammalian nervous system throughout various developmental stages until adulthood. Drawing is not to scale. BF: basal forebrain; CC: cerebral cortex; HIP: hippocampus; ST: striatum; WB: whole brain. (1) Sprague Dawley rat [31]; (2) mouse [32].

At the protein level, members of the JAK-STAT pathway are expressed in different regions of the developing and mature brain, such as the basal forebrain, striatum, hippocampus, and cerebral cortex [31]. Their expression is also found to be differentially regulated, depending on the stages of brain development, and is summarized in Figure 3. JAK1 expression was relatively low compared to JAK2 and its expression was consistent across all developmental stages of rat brain [31]. Expression of JAK2 was higher in the developing brain, specifically at E14 and E18, and gradually diminished towards adulthood [31]. Using Western blot, De-Fraja and colleagues failed to detect expression of JAK3 in selected brain regions as well as in whole brain. Recently, Kim and colleagues [32] found that JAK3 expression was increased in embryonic (E11 and E15) and postnatal brains (P6), but its expression diminished towards adulthood. Expression of another JAK member, TYK2, was not detected in both developing and mature brain [31].

Activated JAK members trigger expression of STAT proteins. STAT1 has a complex expression pattern that varies at different brain developmental stages. STAT1 expression levels have been shown to gradually decrease from E14 to P2, with an increase in expression in adulthood [31]. Later in the aging brain (26 months), expression of STAT1 remained invariant [33]. STAT3 was found to be constitutively expressed in the cerebral cortex and the hippocampus of both embryonic and adult brains. Its expression in the striatum and the basal forebrain was higher at E14 and gradually decreased from E18 towards adulthood [31]. STAT3 is needed for pleiotropic action, such as determination of neuronal cell fate, survival, regeneration, and apoptosis throughout brain development. In 2000, De-Fraja and colleagues observed that expression of STAT3 was markedly downregulated in the aging brain (26 months) [33].

In general, expression of STAT5 is low in the cortex and basal forebrain of immature brain, and its expression becomes gradually more pronounced towards adulthood. Interestingly, STAT5 in the striatum showed a reverse expression pattern. Furthermore, STAT5 has been shown to be weakly expressed in the hippocampus in both embryonic and postnatal brains [31]. STAT6 has been shown to be consistently expressed across all brain regions throughout the embryonic stages (E14–E18). Its expression then progressively decreased in more developed stages (P2, P10, and adult) [31]. The STAT4 protein, however, was not detectable in any brain region (cerebral cortex, striatum, basal forebrain, and hippocampus) [31]. Therefore, JAK-STAT mRNA and proteins are spatiotemporally expressed and function in the regulation of neurodevelopment in both developing and mature brain.

## 4. The Role of JAK-STAT Pathway in Neuronal Differentiation and Gliogenesis

JAK-STAT signaling is essential for gliogenesis, rather than promoting neurogenesis. During the neurogenic phase, JAK-STAT signaling is tightly regulated by DNA methylation; however, JAK-STAT pathway activity becomes robustly activated during the transition from neuronal to glial differentiation. Moreover, various intrinsic and extrinsic gliogenic signals dictate neuroepithelial cells to switch from a neuronal to glial differentiation, such as epigenetic signals and transcription factors (intrinsic) as well as cytokines and growth factors (extrinsic) [34].

In the canonical JAK-STAT signaling pathway, cardiotrophin-1 binds to gp130 and LIF*β* coreceptors and activates the JAKs. The STAT3 transcription factor is then activated through phosphorylation by JAKs. Active STAT3 then binds the p300/CBP coactivator proteins and forms a larger complex with Smad, which is a downstream effector of bone morphogenic protein signaling [35]. This Smad:p300/CBP:STAT3 complex then translocates into the nucleus, which specifies glial cell fate by transcriptional activation of astrocytic genes, such as* Gfap* and* S100β*. However, STAT3 can also be activated by different ligands, such as IFN and interleukins (Table 1) [26, 36–38].

**Figure 4:**
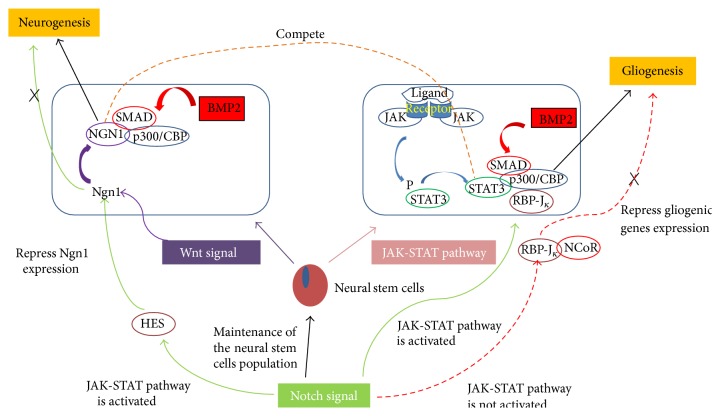
Crosstalk between Wnt, Notch, and JAK-STAT signaling pathways. Wnt signaling activates Ngn1, which then binds p300/CBP coactivator proteins to promote neuronal differentiation. Ngn1 competes with STAT3 to inhibit gliogenesis during neurogenic phases. During gliogenesis, the STAT3 transcription factor is activated through phosphorylation by JAKs. The active form of STAT3 binds the p300/CBP coactivator proteins and promotes gliogenesis. Notch signaling activates gliogenic genes; HES inhibits neurogenesis via inhibition of proneural genes. At the same time, RBP-J*κ* is also activated to promote gliogenesis. RBP-J*κ* binds a repressive cofactor protein, termed nuclear receptor corepressor, to suppress gliogenic genes and inhibit glial cell differentiation when the JAK-STAT pathway is not activated.

Activation of JAK-STAT signaling alone is insufficient to initiate gliogenesis. Other factors that promote gliogenesis, such as Notch signaling, are also required; however, the gliogenic action of Notch signaling must coincide with activation of JAK-STAT signaling (Figure 4). At the same time, neurogenesis must be inhibited via recombination signal sequence-binding protein J*κ* (RBP-J*κ*). RBP-J*κ* binds to the repressive cofactor protein nuclear receptor corepressor to suppress gliogenic genes and inhibit glial cell differentiation when the JAK-STAT pathway is not activated [39].

Epigenetic alteration of chromatin structure by the polycomb group complex during the transition to gliogenesis leads to suppression of* Ngn1* and* Ngn2* and promotes glial differentiation [40]. In addition, Notch effector protein nuclear factor I/A binds to astrocytic gene promoters, such as* Gfap*, to induce dissociation of the DNA methylating enzyme [41]. Consequently, the chromatin enters a relaxed state, allowing for the transcription of gliogenic genes, such as* Gfap*,* Stat1*, and* S100β* [42].

## 5. Potential Roles of JAK-STAT Signaling in Promoting Gliogenesis in DS Brain

The overproduction of several cytokines has been reported to be associated with the pathophysiology of DS. Gliogenic shift in the DS brain may be modulated by the different ligands and receptors that activate the JAK-STAT signal transduction pathway. For example, the level of IFN*γ* is markedly increased in trisomy 16 (Ts16) mouse fetus brain [43]. Together with the overexpression of the IFN receptor gene [44], this may sensitize the cells to interferon interaction and lead to activation of the JAK-STAT signaling pathway.

Gliogenic shift has been observed in both DS human and mouse brains. In the hippocampal region of human fetal DS brains at 17–21 weeks of gestation, a significantly higher number of astrocytes and lower percentage of neurons have been shown [13]. There was also a reduction in proliferating cells in the hippocampal germinal layer and parahippocampal gyrus [13]. The frontal lobe of 18–20-gestational-week-old human DS fetuses also showed a significantly higher number of radial glial cells and mature astrocytes compared to age-matched controls [19]. Furthermore, precursor cells from cerebellar neurogenic regions (external granular and ventricular zones) of human DS fetuses were proliferation-impaired [12]. Briggs and colleagues demonstrated a twofold increment of increase glial lineages in DS iPSC culture [18]. Interestingly, a neurosphere culture of stem/progenitor cells from the subventricular zone of Ts1Cje mice at P84 showed an increase in astrogliogenesis and reduced neurite outgrowth in differentiated neurons when compared to the age-matched controls despite no differences in the pool of neural stem cells [20]. These results suggest that, in early stages, the neural stem cell pool in the brain of the DS mouse model may not differ from their euploid controls but tends to differentiate into glial lineages and defective neurons as the brain matures or regenerates itself in the adult stage.

Efforts to unravel the disrupted molecular mechanisms that lead to DS learning and memory deficits have been carried out in various studies on human samples as well as mouse models. Sturgeon and colleagues postulated on pathways and HSA21-encoded genes and proteins that may cause intellectual disability through a meta-analysis of databases comprising protein-coding genes, human pathways, and protein-protein interactions [45]. Based on their pathway analysis of HSA21 genes, they reported that JAK-STAT is an enriched pathway with HSA21 protein associations, including IFNAR1, IFNAR2, IFNGR2, and IL10RB [45]. Moreover, RT-qPCR of whole brain samples from the DS mouse model Ts1Cje demonstrated overexpression of* Ifnar1*,* Ifnar2*, and* Il10rb* genes [46]. In addition, global gene expression analysis performed on the cerebral cortex, cerebellum, and hippocampus of Ts1Cje mice at four different postnatal time points (P1, P15, P30, and P84) showed* Stat1* upregulation in Ts1Cje cerebellum and cerebral cortex at P84 as confirmed by RT-qPCR and Western blot [47]. Supporting evidence from a bioinformatics analysis using the Database for Annotation, Visualization, and Integrated Discovery also identified JAK-STAT signaling as a major pathway dysregulated in the Ts1Cje cerebral cortex and hippocampus [48]. There was also a significant increase of IFNAR2 proteins in the cerebral cortex of DS fetuses at 19–21 weeks of gestational age [49].

Impairment of neurogenesis and enhancement of glial cell generation in the DS brain may be the main factors contributing to intellectual disability associated with the syndrome. Although the precise mechanisms have not yet been fully defined, it has been proposed that the action of IFNs (IFN-*α*/*β*) is related to trisomy 21 and related phenotypic anomalies [50]. An extra copy of an IFN receptor (IFNR) gene within the triplicated region leads to a 1.5-fold increase in gene expression, which subsequently increases cellular responsiveness to IFNs. These receptors activate JAK-STAT signaling cascades after binding to their ligands. It is posited that IFNs are involved in this mechanism because their receptors (IFNAR1, IFNAR2, and IFNGR2) are located on the extra copy of the genomic segment of chromosome 21 [51].

The JAK-STAT pathway is canonically induced by IFNs [52], which are key mediators of astrogliogenesis in neural stem cells [53]. IFN*γ* treatment of proliferative wild-type murine E14 neurosphere-derived neural precursor cells showed reduced proliferation but upregulated GFAP  and  *β*III-tubulin expression with simultaneous sonic hedgehog and* Stat1* activation [54]. Moreover, IFN*β* treatment of human SH-SY5Y cells and mouse primary cortical neurons was recently shown to negatively regulate brain-derived neurotrophic factor signaling and action via prevention of tropomyosin-related kinase receptor type B activation. IFN*β* activation of JAK-STAT signaling resulted in downregulation of tropomyosin-related kinase receptor type B, which led to a reduction of neurite outgrowth and neuronal differentiation [55]. Results of these* in vitro *studies suggest that the IFNs and IFNRs are inducers of JAK-STAT signaling in driving gliogenesis of neuronal cell cultures. Unfortunately, information on the source of cytokines or IFNs that result in increased gliogenesis in DS subjects remains limited and was not studied or reported in the aforementioned references. Thus, the mechanism of action whether via paracrine or autocrine is not well described. However, IFN and other proinflammatory cytokines are potentially secreted from DS astrocytes, since* in vitro* studies have demonstrated that media collected from DS astroglia cultures exhibit a neurotoxic effect on neural progenitor cells (NPCs) [21]. In 2014, Ling and colleagues suggested that overexpression of IFNR may increase responsiveness to IFN, thus leading to activation of downstream targets, namely, the JAK-STAT signaling pathway [47]. Therefore, overstimulation of JAK-STAT signaling due to overexpression of IFNRs may promote a neural progenitor cell fate toward gliogenic pathways in the DS brain.

The fact that IFNR genes are triplicated and upregulated in DS individuals and mouse models may predispose DS brain to greater IFN sensitivity. Therefore, IFN-JAK-STAT activation in the neurogenic-to-gliogenic shift should be further investigated, since it may represent a potential therapeutic target for preventing and/or reversing this shift in DS individuals.

## 6. Gain- or Loss-of-Function Mutations in JAK-STAT Genes among DS Individuals

To date, the effect of mutations in JAK-STAT genes among DS individuals on the neurogenic-to-gliogenic shift in the brain has not been clearly delineated. Most of the mutations within these genes were investigated in relation to leukemia. DS individuals have 10- to 20-fold higher risk in developing leukaemia [56], which is a disorder that constitutes 60% of all malignancies in DS individuals [57].

Genetic mutations in JAK kinases (except TYK2) have been highlighted in various diseases, including myeloproliferative disorders and cancers. Mutation of the JAK domains can result in gain- or loss-of-function in the activity of the JAK-STAT pathway. Somatic mutations of JAK1 are more prevalent in adult acute lymphoblastic leukaemia (ALL) patients, especially in those with T cell precursor ALL (T-ALL) [58]. However, these JAK1 mutated cases were also reported to have mutations in NOTCH1 [58], which is the favourable prognosis of childhood T-ALL [59]. The reported mutations were missense and some of them were predicted to have the ability to control the kinase activity through destabilising interdomain interactions [58]. Hornakova and colleagues reported that different ALL-associated JAK1 mutations can differentially potentiate responses to type I interferons [60]. They also showed that an* in vivo* leukaemia model with cells expressing a JAK1 mutation was hypersensitive to the antiproliferative effect of type I interferon [60]. The mechanism for this observation may occur through the proliferative potential of STAT5 signaling and the antiproliferative potential of STAT1 signaling in hematopoietic precursor cells [61]. JAK2 mutations, especially the V617F point mutation that causes a constitutively active kinase [62], are implicated in myeloproliferative diseases [61]. Laurence and colleagues have suggested that the V617F mutation alters the interdomain interaction between the kinase and pseudokinase domain, which is needed to control JAKs in their inactivate state [61]. This causes spontaneous autophosphorylation and activation of the mutant kinases in hematopoietic precursor cells, thus preventing cytokine-mediated control of growth and survival [63, 64]. Moreover, this mutation may disrupt the binding of JAK2 protein to suppressor of cytokine signaling 3 (SOCS3), thus allowing JAK2 protein to circumvent the second regulatory constraint [65].

Interestingly, most of the mutations reported within JAK kinases that are associated with DS-related malignancies were gain-of-function mutations. Activating somatic JAK2 mutations were reported in 20% of DS ALL patients and nearly all of the JAK2 point mutations happened at a common site, an arginine-to-guanine residue at position 683 (A683G) [66]. Functional studies on the mutation in murine Ba/F3 cells showed cytokine-independent growth and constitutive activation of JAK/STAT signaling pathway [67]. Another mutation that involved a five-amino-acid deletion within the JH2 pseudokinase domain in JAK2 (JAK2DeltalREED) was found in DS B-ALL patients [68]. It has been suggested that the location of the JAK2 mutation may affect different downstream signaling cascades in a cell-dependent context [69]. The JAK mutations reported in DS patients were also shown to affect myeloid progenitors, suggesting that these mutations are the secondary acquired genetic events in trisomic progenitor cells [69]. Walters and colleagues identified activating JAK3 mutations in a small subset of DS acute megakaryocytic leukaemia (AML) cases [70, 71]. They showed that the* in vivo* cell model expressing the JAK3 mutants demonstrated cytokine-independent growth and a particular JAK3 mutation (A572V) conferred characteristics of megakaryoblastic leukaemia in C57BL/6 mice [70]. Genomic rearrangement of cytokine receptor-like factor 2 (CRLF2) has been reported to be associated with JAK kinases mutations in childhood B-ALL cases, including DS patients [72, 73]. Although various gain-of-function mutations within JAK kinases have been strongly associated with the development of leukemias in DS individuals, none of these events were described in relation to dysregulated brain development and function. These observations, however, suggest that JAK-STAT signaling as an important pathway within the brain that is potentially associated with the proposed model of neurogenic-to-gliogenic shift.

## 7. IFN-JAK-STAT Targeting as a Potential Therapeutic for DS

Modulation of the level of IFNs or expression/phosphorylation of IFNR-JAK-STAT candidates may reduce JAK-STAT signaling activation, leading to the modification of cell fate determination in DS brain development. Mäkelä and colleagues have reported that IFN-JAK-STAT pathway-activated caspase-3 functions in apoptosis [74]. Although their study was not conducted with neural progenitor cells derived from DS brain, it was suggested that IFN affected cell proliferation and survival. Remarkably, neural progenitor cells derived from E17 rat brain treated with IFN*γ* induced phosphorylation of Stat1, which in turn, activated p21, Bcl-2 family proteins, and caspase-3 [74]. These proteins also affect cell proliferation and promote cell death. In contrast, partial knockout of Ifnar2 and Ifngr2 in neuronal cultures derived from trisomy of chromosome 16 mouse fetuses showed improved cell growth and viability [75]. Moreover, addition of anti-IFN*γ* IgG to the culture medium significantly increased the viability of cortical neurons derived from trisomy of chromosome 16 mouse fetuses but had no effect on euploid neurons [76].

A subset of DS patients exhibit neuropathological features and suffer from early onset Alzheimer's disease (AD). IFN has been shown to activate JAK-STAT signaling, and its proinflammatory effect caused neuronal cell death in AD [77]. Therapeutically, it was suggested that the progression of AD can be reduced by blocking IFNAR1 [77]. Therefore, administration of IFN antagonists may have a therapeutic benefit in DS. Collectively, inhibiting both IFNs and their receptors can improve the viability of neurons and may restore neurogenesis in the DS brain.

The IFN inhibitor Normferon was developed by Dr. Maroun following the positive outcome of targeting IFN and INFR in a DS mouse model [75, 76, 78]. In contrast to IFN inhibitors, there are currently more than ten JAK inhibitors currently being assessed in clinical trials, such as ruxolitinib, SAR302503, lestaurtinib, CYT-387, pacritinib, LY2784544, XL019, AZD1480, NS-018, and BMS-911543 [79]. These drugs were tested in hematologic cancers, such as leukemia and myelodysplastic syndrome. Ruxolitinib, a selective JAK1 and JAK2 inhibitor, is the only JAK inhibitor approved by the US Food and Drug Administration and is mainly used for patients with myelofibrosis. IFN is known to activate JAK1 and JAK2 kinases, which subsequently activate STAT1 [80]. Therefore, inhibitors of JAK1 or JAK2 kinases, such as ruxolitinib, may serve as a potential treatment for DS to restore neurogenesis. Laboratory analysis of low-level administration of ruxolitinib should be carried out to ascertain the effect on neurogenesis in DS mice and/or humans.

Another potential therapeutic for DS is nucleic acid-based therapy. The nucleic acid/oligonucleotide can be antisense, ribozymes, short interfering RNA, microRNA, and aptamers that inhibit gene expression and function at transcriptional or translational levels [81]. Currently, use of the nucleic acid-based approach for targeting STAT focuses on cancer treatment and therapy [82]. However, several limitations have been reported, mainly regarding degradation of the nucleic acid/oligonucleotide when delivered into biological systems [82]. Therefore, chemical modification of the nucleic acid/oligonucleotide, termed locked nucleic acid (LNA), has been proposed to increase its stability. The LNA name stems from the ribose ring of the nucleic acid/oligonucleotide being locked by a methylene linkage between the 2′-oxygen and 4′-carbon [83]. Therefore, incorporating LNA can increase resistance of the nucleic acid/oligonucleotide to nuclease degradation. Its low toxicity also makes it as a potential therapeutic tool [84]. LNA-based nucleic acid targeted-inhibition has specifically emerged as an important platform for drug development. A clinical trial conducted using LNA-antisense oligonucleotide EZN-2968, which targets hypoxia inducible factor-1 (HIF-1), found that overexpression of HIF-1 in cancer cells leads to upregulation of genes important for cancer cell survival [85]. Two out of 10 patients were found to have reduced expression and levels of HIF-1*α* mRNA and protein, respectively, after administration of EZN-2968. A similar strategy may be applied to target the IFN-JAK-STAT signaling pathway for DS therapy by inhibiting expression of IFNRs or JAK-STAT. For example,* miR-9* was shown to directly target mRNAs of* Lif*,* gp130*, and* Jak1* by downregulating these crucial upstream elements of the JAK-STAT signaling pathway, thus leading to decreased phosphorylation of STAT and suppression of astrogliogenesis [86]. Therefore, a* miR-9* LNA mimic could be developed into a potential therapy to regulate the level of JAK-STAT activation. JAK-STAT signaling, however, has pleiotropic functions: inhibiting expression of JAK-STAT may improve the DS phenotype, at least short-term, while it may also cause secondary adverse effects on other organs or systems. Therefore, careful experimental design and result interpretation are crucial for developing an effective targeted therapy for DS individuals.

## 8. Concluding Remarks

JAK-STAT signaling is one of the most important pathways determining gliogenic cell fates. In this review, the potential role of JAK-STAT signaling in neurological diseases, such as DS, has been highlighted. It is believed that neuropathological and cognitive impairment in DS patients may be attributed to, at least in part, defective neurogenesis and a reduction in the number of neurons in several brain regions, including the cerebrum, hippocampus, and cerebellum. In addition, the increased number of astrocytes in DS brain may also be a potential factor leading to intellectual disability in DS individuals.

The bias of neuroepithelial cells towards gliogenesis also indicates dysregulation of JAK-STAT signaling during brain development. The Notch signaling pathway coincides with the JAK-STAT pathway to bring about the gliogenic shift. Without JAK-STAT signaling, the Notch pathway instead represses gliogenic genes. Defective JAK-STAT signaling may contribute to the overproduction of glial cells. Therefore, it is crucial to understand the role of JAK-STAT signaling pathways in controlling astrocytic fate in DS as a potential therapeutic target for improving cognitive function in DS individuals.

## Figures and Tables

**Table 1:** Cytokines and their corresponding specific receptors in JAK-STAT signaling activation.

	Ligands	Receptor	JAK kinases	STATs
IFN family	IFN-*α*/*β*	IFNAR	JAK1, TYK2	STAT1, STAT2, STAT3, STAT5a/5b
	IFN-*γ*	IFNGR	JAK1, JAK2	STAT1, STAT3, STAT5a/5b
	IL-10	IL-10R	JAK1, TYK2	STAT1, STAT3

gp 130 family	IL-6	gp130	JAK1, JAK2	STAT1, STAT3
	IL-11	gp130	JAK1	STAT1, STAT3
	IL-12	IL-12R	JAK2, TYK2	STAT4
	CNTF	gp130 and LIFR*β*	JAK1, JAK2	STAT1, STAT3
	LIF	gp130 and LIFR*β*	JAK1, JAK2	STAT1, STAT3
	OSM	gp130 and OSMR	JAK1, JAK2	STAT1, STAT3
	CT-1	gp130 and LIFR*β*	JAK1, JAK2	STAT3
	G-CSF	G-CSFR	JAK1, JAK2	STAT3
	Leptin	LEPR	JAK2	STAT3

*β*c family	IL-3	IL-3R	JAK2	STAT5a/5b
	IL-5	IL-5R	JAK2	STAT5a/5b
	GM-CSF	GM-CSFR	JAK2	STAT5a/5b

*γ*-chain (gC) family	IL-2 IL-7 IL-9 IL15	IL-2R IL-7R IL-9R IL-15R	JAK1, JAK3	STAT1, STAT3, STAT4, STAT5a/5b
	IL-4	IL-4R	JAK1, JAK3	STAT6
	IL-13	IL-13R	JAK1, JAK2, TYK2	STAT6
